# Periocular Endocrine Mucin-Producing Sweat Gland Carcinoma and Mucinous Carcinoma—An Institutional Series Including 49 Patients

**DOI:** 10.3390/dermatopathology13040048

**Published:** 2026-10-03

**Authors:** Faith Stone, Ashley Campbell, Fatemeh Rajaii, Nicholas Mahoney, Jaroslaw Jedrych, Charles Eberhart

**Affiliations:** 1 Department of Pathology, Johns Hopkins University School of Medicine, Baltimore, MD 21287, USA; 2 Department of Ophthalmology, Johns Hopkins University School of Medicine, Baltimore, MD 21287, USA; 3 Department of Dermatology, Johns Hopkins University School of Medicine, Baltimore, MD 21287, USA

**Keywords:** endocrine mucin-producing sweat gland carcinoma, mucinous carcinoma, MYB, bilateral

## Abstract

Endocrine mucin-producing sweat gland carcinomas (EMPSGCs) are rare adnexal tumors with a predilection for the periocular region, which can progress to primary mucinous carcinoma (MC). Understanding of the clinical and histopathologic features of these carcinomas is still evolving. We present a single-institution series of 45 EMPSGC in 38 patients, and 20 MC in 11 patients arising in the eyelids and periocular region. EMPSGC and MC patients had similar mean ages at presentation (67 and 58), and both were more common in women. One EMPSGC involved bilateral eyelids at presentation, and both tumor types recurred in some cases, but no extensive invasion or metastatic disease was identified. In five MC patients, microscopic examination identified EMPSGC precursor regions within the lesion, while these were absent in the remaining six. Immunohistochemical markers of neuroendocrine differentiation varied over the study period, with INSM1 and synaptophysin used most frequently. INSM1 showed stronger and/or more extensive immunoreactivity in most, but not all, cases. All five patients with EMPSGC-associated MC had tumors positive for a neuroendocrine marker. Finally, all three of the tested EMPSGCs expressed MYB based on RNA in situ hybridization. Our findings support the female predominance in these tumors and their potential for multicentric or bilateral disease at presentation, as well as the capacity of EMPSGC to progress to MC.

## Simple Summary

Endocrine mucin-producing sweat gland carcinoma and mucinous carcinoma are rare skin cancers that most commonly arise on or near the eyelids in older adults. These tumors are thought to be related, with the former potentially progressing to the latter over time, but their relationship is not fully understood due to their rarity. We studied 65 tumors from 49 patients at our institution and found that both tumor types occurred more frequently in women and behaved in a non-aggressive manner, with no spread to distant sites. Nearly half of the mucinous carcinomas contained regions resemble the precursor tumor, supporting a progression pathway. We also found that a protein marker called INSM1 was particularly useful for diagnosis, detecting tumors that other commonly used markers missed. One patient had tumors involving both eyelids simultaneously, highlighting the possibility of multifocal disease.

## 1. Introduction

Endocrine mucin-producing sweat gland carcinoma (EMPSGC) and primary mucinous carcinoma (MC) of the skin are both appendageal tumors arising in older patients, with a predilection for the eyelid and other periocular tissues. The World Health Organization Classification fifth edition includes them in the malignant apocrine and eccrine tumors section, but EMPSGC and MC generally behave in a low-grade fashion, and while they can reoccur locally it is rare for them to metastasize [1,2]. Many of their histopathological features overlap, and EMPSGC has been proposed as a precursor of MC [3]. However, while EMPSGC shows at least focal expression of neuroendocrine markers, this has been reported in only a subset of MC, and the precise relationship between the two tumors is not fully defined.

EMPSGC typically present as slow-growing, painless nodules or cystic lesions in older adults, and in most series are more common in women than in men [3,4,5,6]. Histologically, EMPSGC are well-demarcated overall and can contain cystic, cribriform, solid and papillary regions. Tumor cells are cytologically relatively bland with round to oval nuclei, variable neuroendocrine differentiation, and mucin present in either the neoplastic cell cytoplasm or adjacent extracellular spaces.

Primary MC of the skin also presents in older patients, but without a clear sex predominance in one meta-analysis including 159 cases [7]. They are generally more poorly circumscribed than EMPSGC. Microscopic analysis shows nests and cords of tumor cells floating in lakes of mucin extending through tissue. Not all MC show expression of neuroendocrine differentiation markers. Their appearance can be identical to tumors in the breast and gastrointestinal tracts, and the possibility of metastatic disease should be evaluated by systematic work-up in lesions lacking an in situ component [1,2].

EMPSGCs can be precursors of MC, but both are relatively rare, and only a few large case series have been published, making it hard to establish firm clinicopathological correlations or to assess the relationship between the two [5,8]. Single tumor specimens can contain both well-circumscribed in situ EMPSGC surrounded by myoepithelial cells in some regions and invasive MC in others [3,5]. EMPSGCs have also reportedly recurred locally as MC, supporting the possibility of progressive disease [5].

Molecular analysis holds potential to help define the relationship between EMPSGC and MC, and to identify possible therapeutic targets. However, relatively few genetic studies have been performed and no recurrent drivers have been reported [4]. In one recently reported patient with both EMPSGC and MC, identical missense variants were reported within *ATRX*, but the changes were not clearly pathogenic and their frequency suggested they were germline rather than somatic events [9].

While most EMPSGC and MC are solitary at presentation, multifocal cases, including some involving bilateral eyelids, have been reported [6,10,11,12,13,14]. Some of these were diagnosed as EMPSGC at some sites and MC at others. Asynchronous involvement of multiple sites on the face has also been documented. It has been suggested that multicentric cases such as this may represent a cancer “field effect” [9].

Both EMPSGC and MC must be distinguished from other primary cutaneous neoplasms that show neuroendocrine or mucinous differentiation, including Merkel cell carcinoma, mucinous basal cell carcinoma and low-grade neuroendocrine carcinoma of the skin. Merkel cell carcinoma can also present on the face, but are fast-growing, densely cellular and pleomorphic, show distinctive immunohistochemical features, and are often associated with Merkel cell polyomavirus expression [1,15,16]. The recently proposed low-grade neuroendocrine carcinoma of the skin, also known as sweat gland carcinoma with neuroendocrine differentiation (SCAND), is less well known but could be harder to differentiate from EMPSGC in small biopsies [17,18]. SCAND reported to date all arose on the trunk, are most common in older men, and are generally more infiltrative and clinically aggressive than EMPSGC [18].

Here we present an institutional series of EMPSGC and MC with a description of both clinical and pathological features, including a number of cases highlighting the ability of EMPSGC to progress to MC. Our series also includes a patient with bilateral eyelid EMPSGC at presentation. Finally, as suggested in several recent reports, we found insulinoma-associated protein 1 (INSM1) to be an effective neuroendocrine marker on immunohistochemical analysis [19,20,21].

## 2. Materials and Methods

### 2.1. Study Design

This retrospective case series adheres to the Declaration of Helsinki and was approved by the Johns Hopkins Hospital Institutional Review Board. Institutional pathology archives were searched to identify all internal or consultation cases in the eyelids or periocular region diagnosed as EMPSGC or cutaneous MC between 1 January 2010 and 31 May 2026. A total of 49 patients were identified, including 11 previously reported, who were retained to provide additional clinical and pathologic context within the expanded institutional cohort [4].

A number of patients had more than one specimen included in the study. Separate surgeries for residual or recurrent disease in the same patient were recorded as individual specimens. Multiple specimens were available in five patients with EMPSGC, including four patients with two specimens (three collected at different time points and one collected concurrently) and one patient with three specimens collected at different time points. The patient with concurrent lesions presented with tumors on bilateral eyelids, and these were treated as unique lesions. One patient with EMPSGC-associated MC had two specimens collected at different time points. Among patients with MC, one had seven specimens collected at different time points including three resected between 1993 and 2009. Additional earlier specimens from this patient were mentioned in the clinical record but were unavailable for inclusion in the study. A second MC patient had three specimens collected at different time points.

Clinical and pathologic data were obtained through a review of the electronic medical record. A combination of radiologic and systemic evaluations obtained within 12 months of diagnosis—including computed tomography (CT), magnetic resonance imaging (MRI), positron emission tomography (PET), mammography, and other relevant studies—were reviewed when available to support the classification of tumors as primary cutaneous neoplasms. Owing to the retrospective nature of the study, the availability of clinical information and ancillary studies varied among patients, particularly for consultation cases. Consequently, definitive exclusion of an extracutaneous primary malignancy was not possible in two consultation specimens, although they were negative for TTF1 and mammaglobin, and neither had available evidence supporting metastatic disease.

Cases were categorized into three groups: (1) EMPSGC, (2) EMPSGC-associated MC containing both EMPSGC and MC components; and (3) primary cutaneous MC without histologic or documented clinical evidence of an associated EMPSGC component.

### 2.2. Histopathological Analysis

All available hematoxylin and eosin (H&E) and immunohistochemical slides were reviewed by FS and CE. Faculties of dermatopathology, head and neck pathology and breast pathology were also consulted in a subset of cases. Immunohistochemical stains and in situ hybridization were performed in the Johns Hopkins Hospital Surgical Pathology laboratory using standard clinical protocols and the following reagents: AE1/AE3 (Pancytokeratin clone AE1/3), Ventana, Tucson, AZ, USA (Cat. No. 760-2595) prediluted; Ber-EP4, Dako, Carpinteria, CA, USA (Cat. No. M0804) dilution 1:30; CAM5.2, Cell Marque, Rocklin, CA, USA (Cat. No. 334M-88) prediluted; Chromogranin A, Ventana, (Cat. No. 760-2519) prediluted; CK20 (Cytokeratin 20) Cell Marque, (Cat. No. 334M-88) prediluted; CK7 (Cytokeratin 7) Dako, (Cat. No. M7018) dilution 1:500; EMA (Epithelial Membrane Antigen) CellMarque (Cat. No. 247M-98) prediluted; ER (Estrogen Receptor) Ventana, (Cat. No. 790-4325) prediluted; GATA3, BioCare Medical, Pacheco, CA, USA (Cat. No. CM405B) dilution 1:100; GCDFP (Gross Cystic Disease Fluid Protein) BioLegend, San Diego, CA, USA (Cat. No. 915101) dilution 1:200; INSM1 (Insulinoma-associated protein 1) Santa Cruz Biotechnology, Dallas, TX, USA (Cat. No. sc-271408) dilution 1:200; Ki-67, Roche/Ventana, Indianapolis, Indiana, USA (Cat. No. 790-4286) prediluted.; MYB-ISH (Myeloblastosis in situ hybridization) Advanced Cell Diagnostics (ACD), Newark, CA, USA (Cat. No. 419708); NSE (Neuron Specific Enolase) Ventana (Cat. No. 760-4786) Prediluted; p40: BioCare Medical (Cat. No. AC13066C) dilution 1:100; p63, BioCare Medical, (Cat. No. PMA163AA) prediluted; PR (Progesterone Receptor) Roche/Ventana, (Cat. No. 790-4296) prediluted; SMA (Smooth Muscle Actin) Ventana (Cat. No. 760-2833) prediluted; Synaptophysin, Novocastra (Leica Biosystems), Deer Park, IL, USA (Cat. No. NCL-L-SYNA) dilution 1:400; TTF1 (Thyroid Transcription Factor-1) Ventana, (Cat. No. 790-4398) prediluted. Because IHC studies were performed as part of routine clinical care over the extended study period, antibody panels were not standardized across the cohort and varied between cases.

EMPSGCs were diagnosed as described in the WHO fifth edition, with the essential criteria including solid, papillary, and/or cystic architecture, polygonal cells with low mitotic activity, mild cytological pleomorphism, neuroendocrine nuclei, and intracellular or extracellular mucin, while expression of neuroendocrine markers was considered a desirable criteria [1,2]. The diagnosis of mucinous carcinoma also used WHO criteria, with tumors composed of nests and strands of atypical epithelial cells floating in lakes of extracellular mucin considered essential [1,2].

Mucinous carcinoma containing well-circumscribed EMPSGC-like regions with limited or absent mucin, or in situ components contained within ducts with a preserved peripheral p40, p63 or smooth muscle actin (SMA) positive basal/myoepithelial cell layer, were designated as EMPSGC-associated MC. The presence of an in situ component was also used to exclude a metastatic MC, and in its absence this was addressed when possible by clinical investigation.

Neuroendocrine differentiation was defined as immunoreactivity for at least one available neuroendocrine marker, including insulinoma-associated protein 1 (INSM1), synaptophysin, chromogranin, or neuron-specific enolase (NSE). Additional markers including ER, PR, cytokeratin AE1/AE3, CAM5.2, CK7, BerEP4, GCDFP-15 and GATA3 were also evaluated when possible. Finally, RNA in situ hybridization for MYB was performed in some recent cases.

### 2.3. Data Analysis

Deidentified data were compiled and analyzed using Microsoft Excel Version 2608 (Microsoft Corp., Redmond, WA, USA). 

## 3. Results

### 3.1. Patient Demographics

A total of 65 specimens were identified from 49 patients, including 45 EMPSGCs in 38 patients, six EMPSGC-associated MCs in five patients, and 14 cutaneous MCs without documented clinical or histopathological evidence of EMPSGC or a mucinous carcinoma at another site in six patients (Table 1). Clinical follow-up was available for 15 patients, with a median follow-up duration of 47 months (range, 4–348 months).

The EMPSGC cohort demonstrated a marked female predominance, with 31 women and seven men (female-to-male ratio, 4.4:1). In contrast, EMPSGC-associated MC arose almost equally among women and men (three women and two men). Finally, pure MC were identified in four women and two men. The mean age at diagnosis was 67 years for both EMPSGC and EMPSGC-associated MC, and 58 years for pure MC.

Race and ethnicity data were available for a subset of patients (Table 1). Among patients with available information, most were White, followed by Black/African American and Hispanic/Latino patients. The percentage of MC patients who were Black/African American patients was relatively high, but the overall numbers were small. Full demographic data were unavailable for a substantial proportion of cases, including most consultations.

### 3.2. Clinical Features

Tumors most frequently involved the eyelids (Table 2). Among EMPSGC cases, the lower eyelid was the most common site (25/45, 56%), followed by the upper eyelid (12/45, 27%). In contrast, EMPSGC-associated MC were most common in the upper eyelids (5/6, 83%). Pure MC were more varied in their location, but also frequently involved the upper eyelids (5/14, 36%) and periocular region including the medial and lateral canthi, temple and cheek (6/14, 43%).

Clinical presentation was variable and included slowly enlarging eyelid nodules, cystic lesions, papules, plaques, and localized eyelid swelling. Preoperative clinical impressions frequently favored benign adnexal lesions including cysts, or more common periocular malignancies such as basal cell carcinoma. Many cases were simply designated “lesion” or “benign lesion”. EMPSGC and MC were not leading clinical concerns preoperatively in any patient at initial presentation. Surgical management primarily consisted of local excision, sometimes following an initial biopsy, or Mohs micrographic surgery.

Several notable clinical presentations were observed. One patient presented with synchronous bilateral EMPSGC in the left and right lower eyelids. Another experienced eight documented local recurrences of MC over 29 years, with the first diagnosed at the Armed Forces Institute of Pathology as MC. No changes in histopathological appearance or spread outside the periocular region were noted in any of these recurrences. Three patients had other neoplasms documented in the clinical record. The first was diagnosed with adenocarcinoma of the colon several years after his EMPSGC was excised, and the colonic carcinoma eventually metastasized to the lung. A second male patient had a history of lung carcinoid, metastatic papillary thyroid carcinoma, moderately differentiated infiltrating ductal breast carcinoma, multiple dysplastic nevi, melanoma, basal cell carcinoma, mycosis fungoides and cutaneous neurofibroma prior to presentation with EMPSGC. Finally, one female patient whose EMPSGC-associated MC was reviewed in consultation had a reported history of invasive ductal carcinoma of the breast.

### 3.3. Histological Analysis of EMPSGC

**Figure 1:**
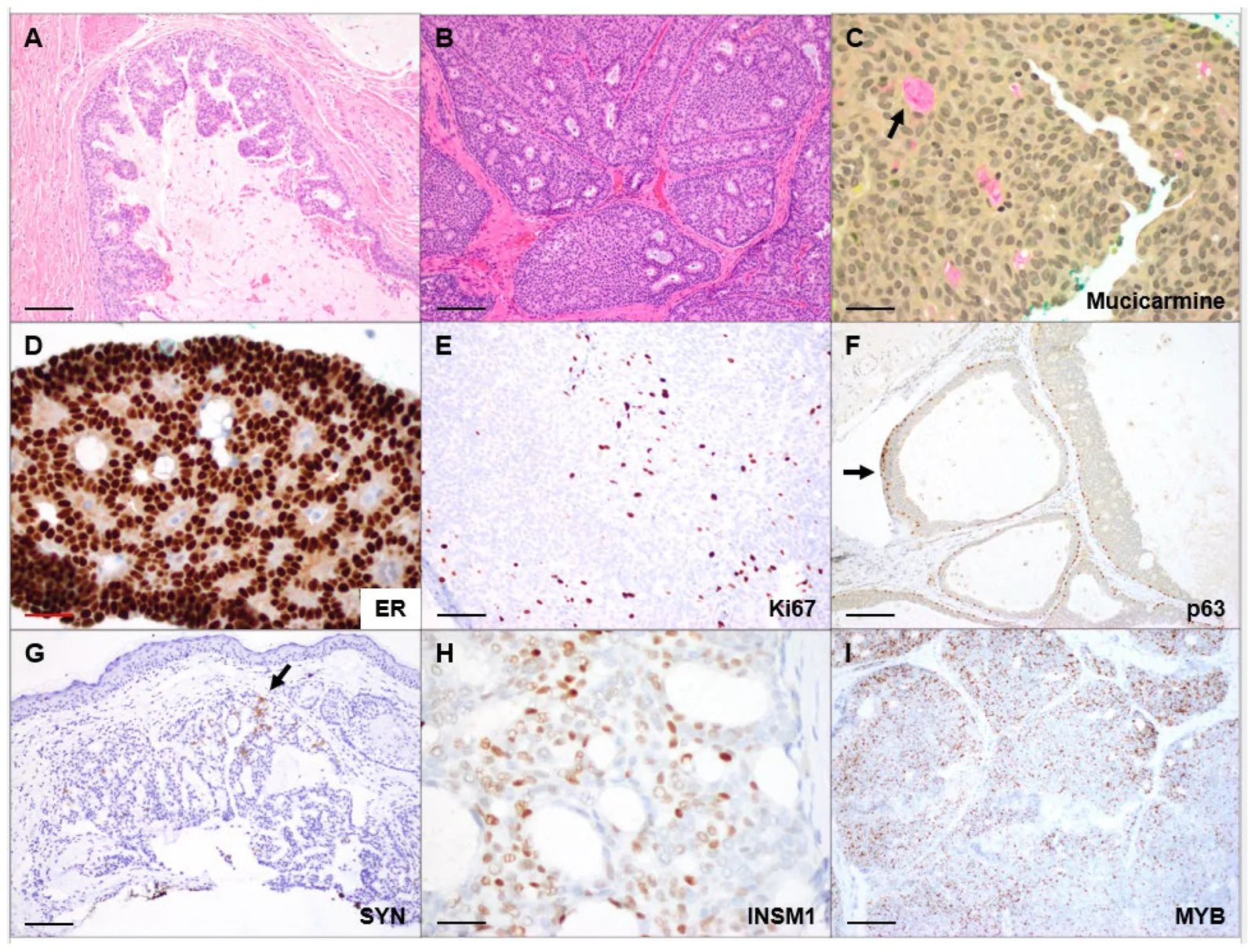
EMPSGC Histopathology: (**A**) Cystic EMPSGC with papillary areas (100×, 100 μm scale bar). (**B**) Solid and glandular/cribriform EMPSGC (100×, 100 μm scale bar). (**C**) Intracellular and extracellular (arrow) mucin deposition (400×, 25 μm scale bar). (**D**) Diffuse, strong nuclear ER expression in EMPSGC cells (400×, 25 μm scale bar). (**E**) Proliferating cells highlighted by Ki67 (200×, 50 μm scale bar). (**F**) Myoepithelial cells positive for p63 surround this in situ EMPSGC (100×, 100 μm scale bar). (**G**) Synaptophysin expression is often focal (arrow, 100×, 100 μm scale bar). (**H**) Nuclear INSM1 expression in EMPSGC cells (400×, 25 μm scale bar). (**I**) MYB expression in tumor lobules identified using RNA in situ hybridization (100×, 100 μm scale bar).

EMPSGC were well circumscribed overall, but demonstrated a spectrum of microscopic patterns, including solid, cystic, papillary and glandular or cribriform growth (Figure 1A,B). Multiple growth patterns frequently coexisted within individual tumors, with solid and cystic elements predominating, and many of the latter containing papillary projections (Figure 1A). The neoplastic cells were cytologically bland, with relatively uniform round to oval nuclei lacking nucleoli. Variable amounts of cytoplasmic and extracellular mucin were present, most prominently in cystic or glandular tumors (Figure 1C). In some cases a solid, cystic or papillary tumor was present within what appeared to be dilated eccrine or apocrine glands or ducts, which has been referred to as in situ EMPSGC [3]. Mitoses were rare, and two cases contained small necrotic foci.

Under immunohistochemical analysis, all 31 EMPSGCs evaluated for cytokeratin 7 (CK7) were positive. ER staining was also diffusely positive in 36 of 36 cases tested (Figure 1D), and PR in 13 of 13. CK20 was negative in the 11 cases for which testing was performed, while the six cases stained for GCDFP15 and/or GATA3 were all positive. The Ki67 proliferation index was relatively low (generally under 10%) in the 13 cases tested (Figure 1E). Most of the 10 EMPSGCs stained for myoepithelial markers including p63, p40 or SMA contained well-defined regions of in situ carcinoma surrounded by positive basal cells (Figure 1F), although two did not.

With respect to markers of neuroendocrine differentiation, 40 of 41 tumors tested were positive for at least one marker (synaptophysin, INSM1, chromogranin or NSE). Of the 37 tumors stained for synaptophysin, 23 showed focal or patchy expression, seven were diffusely positive, three were positive but the extent of staining was not recorded, and four were negative (Figure 1G). Intensity of this staining varied, but most were moderate or strong. Two of the four synaptophysin-negative specimens, representing separate surgical procedures from the same patient, demonstrated focal INSM1 positivity. The other two synaptophysin-negative cases could not be evaluated for INSM1, but one of them was focally positive for NSE. Overall, INSM1 was positive in 11 of the 11 cases tested, but one case showed very focal and faint expression. Chromogranin was positive (almost always focally) in 12 tumors, and negative in nine, with all but two of the latter expressing INSM1 or synaptophysin. Of the six tumors tested for NSE, two were diffusely positive, three were focal, and one was negative. The tumor that was negative was also negative for synaptophysin and chromogranin.

### 3.4. Histological Analysis of EMPSGC-Associated MC

**Figure 2:**
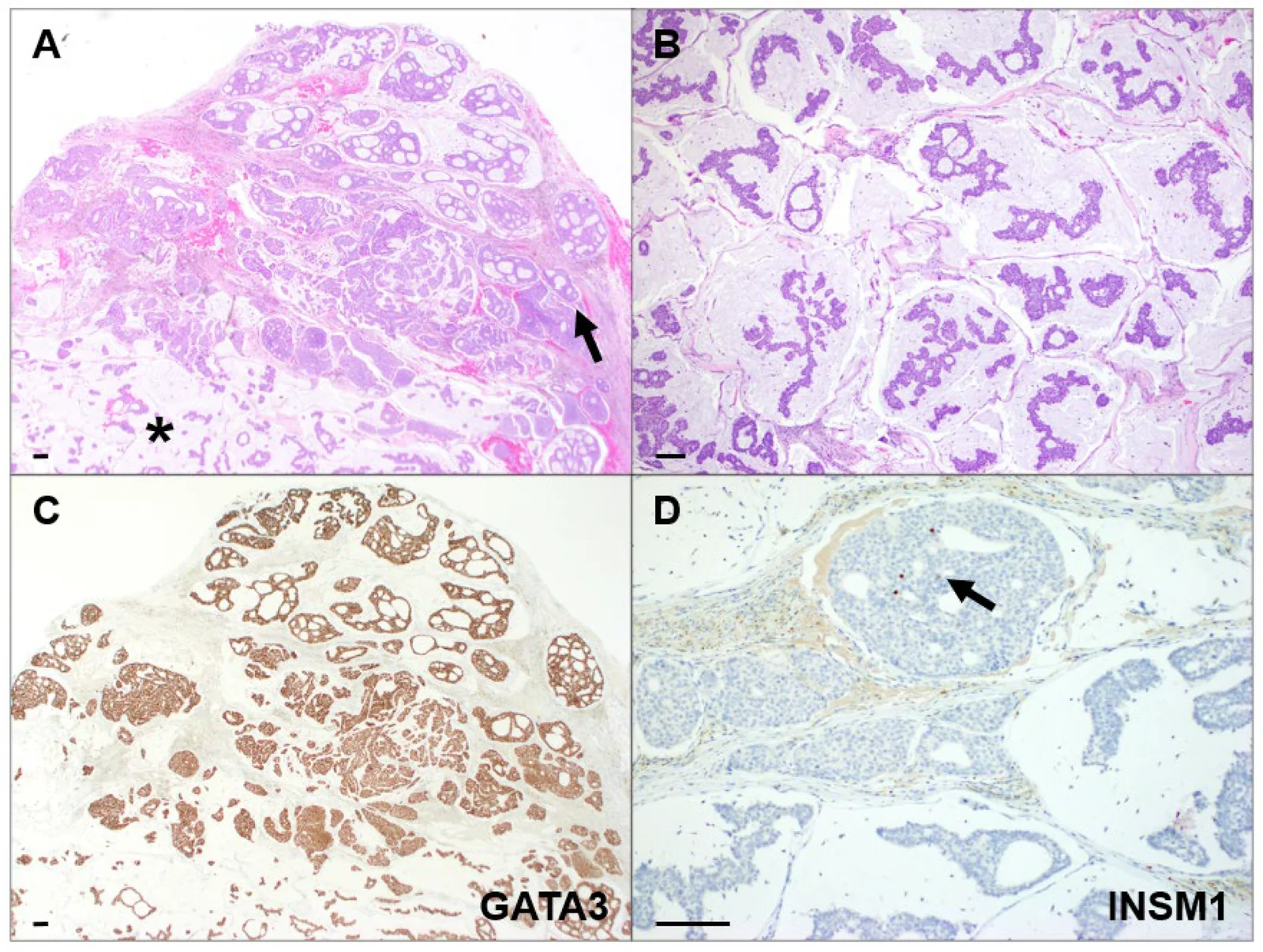
EMPSGC-associated MC Histopathology: (**A**) Tumor with both well-circumscribed EMPSGC (arrow) and diffusely mucinous (asterisk) components (20×). (**B**) Mucinous carcinoma component (40×). (**C**) Diffuse GATA3 expression (20×). (**D**) Very focal INSM1 expression (arrow, 100×). (100 μm scale bars).

EMPSGC-associated MC demonstrated areas of conventional EMPSGC transitioning to mucinous carcinoma (Figure 2A). Invasive tumor was characterized by epithelial nests, clusters, and single cells suspended within extracellular mucin pools (Figure 2B).

Immunohistochemical profiles were similar to those seen in EMPSGC, and of the six cases examined four of four were positive for CK7, 0 of three for CK20, five of five for ER and four of five for PR. The single PR-negative case was positive for ER. Neuroendocrine marker expression was identified in five of six tumors, with three of three positive for INSM1, one of three positive for synaptophysin and one of three positive for chromogranin. The negative tumor, which was biopsied and then excised, lacked synaptophysin in both specimens, with NSE (but not chromogranin) expression in the larger resection. Low-to-moderate proliferation was seen in the single case assessed using Ki67.

### 3.5. Histological Analysis of MC

**Figure 3:**
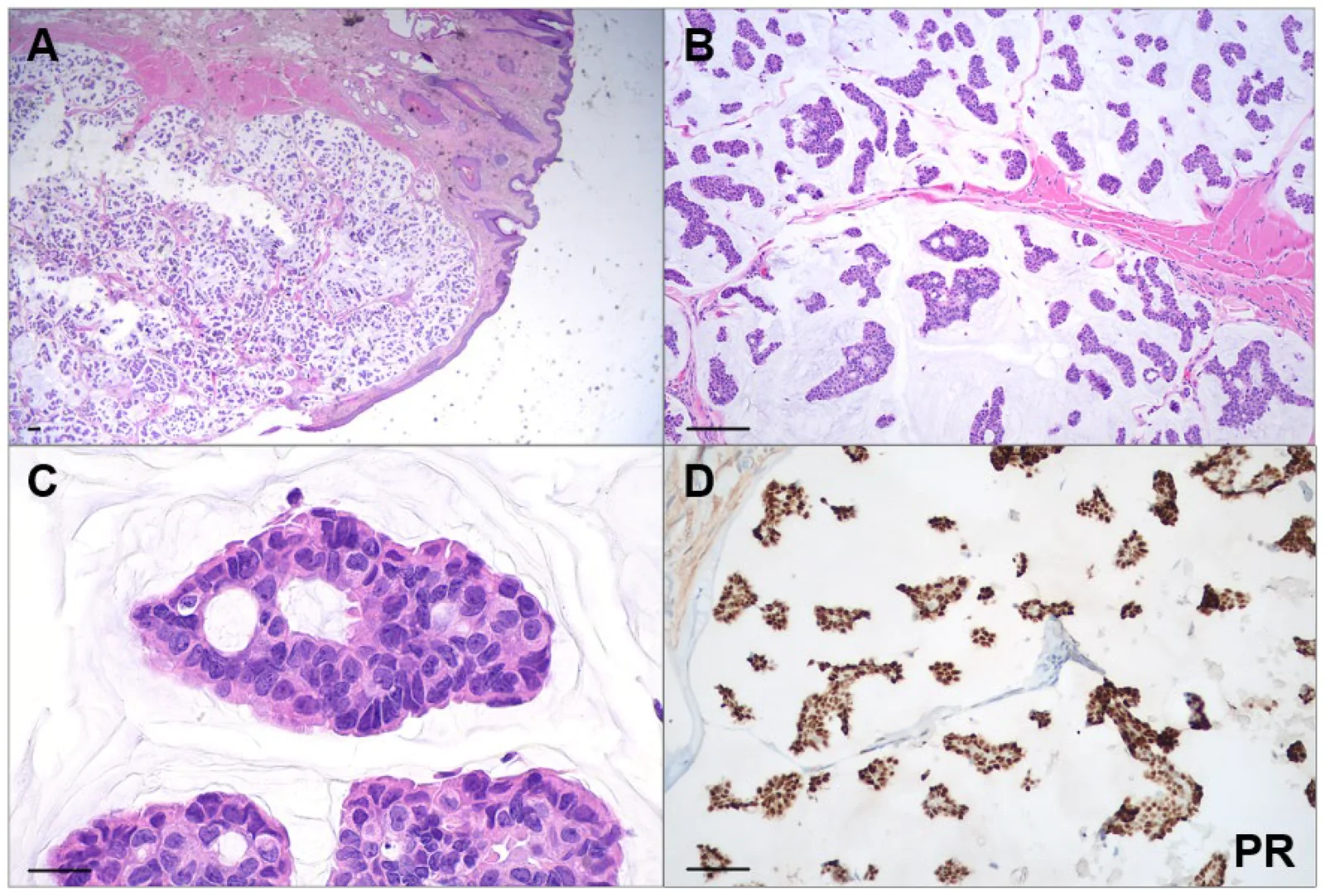
Mucinous Carcinoma Histopathology: (**A**) Mucinous tumor extends diffusely through the eyelid stroma (20×, 100 μm scale bar). (**B**) Nests and strands of carcinoma in pools of mucin (100× 100 μm scale bar). (**C**) Mucinous carcinoma comprising atypical round to oval cells forming small glandular structures (400×, 25 μm scale bar). (**D**) Diffuse PR expression (100×, 100 μm scale bar).

The MC in our series showed nests and strands of atypical epithelial cells floating in extensive pools of extracellular mucin but lacked a concurrent or antecedent EMPSGC or in situ component (Figure 3). They were more poorly circumscribed overall, with mucin dissecting into dermis and deeper tissues. Gland formation was noted in some tumor nests. The overall immunohistochemical profile was similar to that seen in the lesions described above, with expression of ER and/or PR as well as CK7, but not CK20. Neuroendocrine markers were not assessed in the seven MC recurrences in the single patient examined at our institution, but of the remaining tumors one of four were positive for synaptophysin, one of two for INSM1, and one of two for chromogranin. Overall, among the five MC specimens, in which at least one neuroendocrine marker was examined, two were positive.

### 3.6. Clinical Outcomes

Follow-up information was available for 15 patients. Recurrence was most frequently associated with tumors containing an invasive mucinous carcinoma component. No cases of metastatic disease were identified. No disease-related deaths were documented during the available follow-up period.

## 4. Discussion

The demographic and clinicopathologic characteristics of our cohort were largely consistent with prior studies on EMPSGC and MC. EMPSGC demonstrated a marked female predominance and occurred primarily in older adults, with the lower eyelid representing the commonest site of involvement. These findings align with previous institutional series and literature reviews reporting similar sex ratios and mean ages at presentation in the sixth to seventh decades [3,4,5,6,22,23]. The clinical presentation of tumors in our series underscores the diagnostic challenge posed by these rare neoplasms, as preoperative clinical impressions frequently favored benign adnexal lesions or more common periocular malignancies, with EMPSGC and MC rarely considered preoperatively [23].

Our findings provide additional support for the capacity of EMPSGC to progress to MC. Among the 11 patients with MC, five (45%) demonstrated an identifiable EMPSGC component, consistent with prior series reporting concurrent EMPSGC in approximately 40-60% of primary cutaneous MC [3,5]. Whether MC lacking an EMPSGC component arose from unsampled or obliterated precursors, or alternatively represents a distinct pathway of mucinous carcinogenesis, remains uncertain. The case of bilateral synchronous EMPSGC in one patient adds to the growing literature documenting multicentric disease, suggesting that a subset of patients may harbor a field defect predisposing to tumor development across the periocular region [6,9,10,11,12,13,14]. Clinically, patients presenting with EMPSGC or MC may benefit from close examination of the contralateral eyelid and surrounding tissues during diagnosis and surveillance.

The microscopic appearance and immunohistochemical profiles observed were consistent with those previously reported. Diffuse ER and PR expression confirms the hormone receptor positivity characteristic of these tumors, while uniform CK7 positivity and CK20 negativity aligns with an apocrine/eccrine immunophenotype [1,2,3,5]. Preservation of a peripheral myoepithelial cell layer, demonstrated by p63, p40, or SMA positivity, was valuable for identifying in situ components and supporting primary cutaneous origin, although this is not helpful in pure MC.

Neuroendocrine differentiation is a defining feature of EMPSGC. In our series, 40 of 41 (98%) of EMPSGC expressed at least one neuroendocrine marker. Synaptophysin showed positivity in 33 of 37 (89%) tested cases, though expression was often focal, and chromogranin demonstrated even more variable expression with only 12 of 21 (57%) positive tumors. INSM1 emerged as particularly useful, with 11 of 11 (100%) positive including two cases negative for synaptophysin. While only a subset of cases in our study could be tested for multiple markers, overall these findings support the recent reports advocating for INSM1 as a sensitive neuroendocrine marker in EMPSGC [19,20,21].

Notably, neuroendocrine marker expression differed between the tumor categories. Nearly all EMPSGC and EMPSGC-associated MC demonstrated neuroendocrine differentiation, and the single negative specimen was an initial biopsy in a patient whose subsequent resection was focally NSE-positive. This was an older case, and due to its lack of specificity and NSE staining was not evaluated in more recent cases in our series. Only 40% of pure MC showed positivity for a neuroendocrine marker. None of the neuroendocrine-negative MC contained an identifiable EMPSGC component, suggesting that MC retaining neuroendocrine features may be more likely to have arisen from an EMPSGC precursor. However, the small numbers and lack of uniform immunohistochemical analysis in our study warrant caution and more data are needed in this area.

RNA in situ hybridization for MYB was positive in all three EMPSGCs tested. Recent studies have identified MYB protein expression in EMPSGC and primary cutaneous MC [17,24,25]. While our sample size is limited, consistent positivity for MYB mRNA extends the prior immunohistochemical studies.

Clinical outcomes mirrored prior reports, with EMPSGC demonstrating a generally indolent course while tumors containing invasive mucinous carcinoma accounted for most recurrences [3,5,8]. No metastatic disease was identified, reinforcing the favorable prognosis despite the potential for persistent local disease when complete excision is not achieved. However, a significant limitation in our study was the relatively limited number of patients for whom prolonged clinical follow-up was available. With respect to the association of mucinous eyelid tumors and other types of neoplasia, two male patients were diagnosed with other malignancies either before or after their EMPSGC, including one with adenocarcinoma of the colon which metastasized to the lung, and a second with lung carcinoid, metastatic papillary thyroid carcinoma, infiltrating ductal breast carcinoma, melanoma, basal cell carcinoma, mycosis fungoides and cutaneous neurofibroma. Finally, a female patient with EMPSGC-associated MC had a reported history of invasive ductal carcinoma of the breast. These cases provide some support for the concept that well-differentiated cutaneous mucinous neuroendocrine tumors can be associated with broader germline predisposition to cancer, including breast cancer in both men and women [6,21,26,27]. However, the number of patients in our series with multiple tumors is relatively small and should be viewed in the context of overall malignancy prevalence in the general population.

## 5. Conclusions

In conclusion, this institutional series provides clinicopathologic evidence supporting the capacity of EMPSGC to progress to primary cutaneous MC. Our findings confirm the female predominance, periocular predilection, and potential for bilateral disease. They also suggest an overall favorable prognosis of these tumors in patients for whom follow-up is available. INSM1 appears to be a sensitive neuroendocrine marker, particularly useful when other markers are equivocal. MYB expression warrants further investigation as both a diagnostic marker and potential therapeutic target. Recognition of the clinicopathologic spectrum of EMPSGC and MC is essential for accurate diagnosis and appropriate management.

## Figures and Tables

**Table 1:** Patient demographics (n = patients).

	EMPSGC (n = 38)	EMPSGC-Associated MC (n = 5)	MC (n = 6)
*Sex*			
Women	31	3	4
Men	7	2	3
*Average Initial Age*	67	67	58
*Race/Ethnicity*			
White	11	2	1
Black American/ Caribbean/African	2	2	2
Hispanic/Latino	1	0	0
Unknown	24	1	3

**Table 2:** Tumor locations (n = specimens).

	EMPSGC (n = 45)	EMPSGC-Associated MC (n = 6)	MC (n = 14)
Right Upper Eyelid	4	3	1
Right Lower Eyelid	13	1	2
Right Undesignated Eyelid	2	0	0
Right Periocular	3	0	6
Left Upper Eyelid	8	2	4
Left Lower Eyelid	12	0	0
Left Undesignated Eyelid	1	0	1
Left Periocular	2	0	0

## Data Availability

All data from this study is provided within the manuscript.

## Abbreviations

The following abbreviations are used in this manuscript:

CK7/20	Cytokeratin 7/20
EMA	Epithelial Membrane Antigen
EMPSGC	Endocrine mucin-producing sweat gland carcinoma
ER	Estrogen Receptor
GATA3	GATA binding protein 3
GCDFP-15	Gross cystic disease fluid protein 15
IHC	Immunohistochemistry
ISH	In situ hybridization
MC	Mucinous carcinoma
MYB	Myeloblastosis
NSE	Neuron specific enolase
PR	Progesterone Receptor
SMA	Smooth muscle actin
TTF1	Thyroid transcription factor 1
